# Femoral Notch Anatomy and Tibial Slope in Anterior Cruciate Ligament Injuries: An Observational Study

**DOI:** 10.31729/jnma.9144

**Published:** 2025-07-31

**Authors:** Jayant Bhagwan Mishra, Sunil Singh Thapa, Kaushal Raj Kafle, Sundar Suwal, Arjun Prasad Lamichhane

**Affiliations:** 1 Department of Orthopedics and Trauma Surgery, Tribhuvan University Teaching Hospital, Maharajgunj, Kathmandu, Nepal; 2 Provincial Hospital, Kalaiya, Bara, Nepal; 3 Department of Orthopedics, Nepal Armed Police Force Hospital, Balambu, Kathmandu, Nepal; 4 Department of Radiology, Tribhuvan University Teaching Hospital, Maharajgunj, Kathmandu, Nepal

**Keywords:** *anatomy*, *anterior cruciate ligament injuries*, *risk factors*

## Abstract

**Introduction:**

The anterior cruciate ligament is a primary knee stabilizer frequently injured during sports activities, with risk factors including intrinsic anatomical variations. These anatomical factors vary by race, sex, and body structure. The study aimed to identify anatomical risk factors for anterior cruciate ligament injury in the Nepalese population using magnetic resonance imaging.

**Methods:**

This observational comparative study was conducted from December 2021 to July 2023 after obtaining ethical clearance (Reference number: 286(6-11) E2 078/079). Magnetic resonance imaging scans of 122 knees (61 anterior cruciate ligament injured and 61 intact) were analyzed. Intercondylar femoral notch width, notch width index, notch angle, medial and lateral posterior tibial slopes were measured. Notch shapes (A, U, W) were classified. Independent t-test and chi-square test were used for comparing continuous and categorical variables, respectively. The Statistical Package for the Social Sciences (version 26) was used for analysis.

**Results:**

The anterior cruciate ligament injured group showed significantly smaller mean notch widths (coronal: 1.90±0.28cm, axial: 1.98±0.30cm), lower notch width indices (coronal: 0.27±0.03, axial: 0.28±0.04), and more acute notch angles (coronal: 49.89±6.12°, axial: 48.70±6.67°) compared to controls (all p<0.05). Both medial (11.23±5.21° vs 8.76±4.76°) and lateral (11.24±5.33° vs 8.76±4.94°) posterior tibial slopes were significantly steeper in injured knees (p<0.01). Type A notches were more common in injured knees.

**Conclusions:**

Smaller femoral notches, lower notch indices, more acute notch angles, and steeper posterior tibial slopes were associated with anterior cruciate ligament injuries.

## INTRODUCTION

The injury of the anterior cruciate ligament (ACL), a primary knee stabilizer, has been ever increasing with the increasing involvement in athletic activities.^1^ Both anatomical and environmental factor has been attributed to this increased risk.^1-3^ Among many anatomical variation, femoral intercondylar notch morphology and tibial slopes are of particular interest, as differences are observed among races, sexes and age groups.^4-7^

Literature has demonstrated extensive study on the anatomical risk factors for ACL injury using various radiological modalities, yet data on Nepalese population is limited.^2,8^ Besides, conflicting results has always been demonstrated in the parameters like notch shape, notch angles and the medial tibial slope. This gap highlights the utility of population specific study, as morphometric variations may affect the risk of ACL injury.

The aim of this study was to determine the association between femoral notch parameters (width, width index, angle, and shape) and posterior tibial slopes (PTS) (medial and lateral) with ACL injury in a Nepalese population using magnetic Resonance imaging (MRI).

## METHODOLOGY

This analytical cross-sectional study was conducted to evaluate anatomic risk factors in patients with ACL injury using MR images at Tribhuvan University Teaching Hospital, Department of Orthopedics and Trauma Surgery and Department of Radiology. It was conducted from December 2021 to July 2023 after receiving ethical approval from the institutional review committee (IRC) (Reference number: 286(6-11)E2 078/079).

The sample size was calculated using the formula for comparing two means, considering the intercondylar notch angle in the coronal plane as the primary parameter using reference values of mean angles of 57.6° (ACL injured) versus 61.71° (ACL intact) with a standard deviation of 8.05°.^9^

n = [2(zα + zβ)²S²]/d²,

where zα = 1.96 at 95% confidence level,

zβ = 0.84 for 80% power

S represents the standard deviation (using the larger value between groups),

and d is the mean difference between groups

The sample size was calculated to be 60 cases per group.

ACL injuries diagnosed with MRI of age group 18-50 years were taken as case and patients undergoing MRI knee for diagnoses other than ACL injury as control. For both groups exclusion criteria included age <18 or >50 years, multi-ligamentous injury, MRI evidence of previous knee surgery, and history of tibial or femoral condyle fracture.

MRI examinations undertaken with a 3 Tesla with standard sequences including T1-weighted, T2-weighted and proton density weighted images in the axial, sagittal and coronal plane were included in the study. All measurements were performed using RadiAnt DICOM Viewer software (Medixant, Poland) by the principal investigator and was verified by an experienced radiologist.

The anatomical parameters included intercondylar notch angle measured in degrees, notch width (NW) in centimeters, and notch width index (NWI) in axial and coronal planes, along with medial and lateral posterior tibial slopes measured in degrees in the sagittal plane in degrees in the sagittal plane in T2 weighted images.

NW (axial and coronal planes) was measured at its narrowest point at the popliteal groove level, while bicondylar width was the sum of notch width along with the medial and lateral condylar widths measured at the same level. The NWI was calculated as the ratio of notch width to bicondylar width. The angle formed by the lines connecting the lowest points of the medial and lateral condyles to the top of the intercondylar notch in the coronal plane, at the level where ACL and Posterior Cruciate Ligament (PCL) intersected near the mid substance of ACL was taken as the intercondylar notch angle. Posterior tibial slope was measured as per Hashemi et al.’s method using sagittal T2-weighted images, where the angle was formed between a line perpendicular to the tibial longitudinal axis and a line connecting the anterior and posterior cortical margins of the tibial plateau.

Femoral notch morphology was classified as per van Eck et al.’s criteria based on axial images into A, U, and W types.^10^ Type A represented a stenotic notch narrowing from base to apex, Type U showed a wider contour without midsection tapering, and Type W exhibited two apparent apices but morphologically similar to Type U. Type W is also described by others as non-A/non-U type or Ω type.^6^

Statistical analysis was done using independent two-sample t-tests for continuous variables (age, notch parameters, tibial slopes) and chi-square tests for categorical data (notch shape classification). Data analysis was done using SPSS version 26.0 (IBM Corp., Armonk, NY, USA). A p-value of less than 0.05 was considered statistically significant.

## RESULTS

Seventy-four patients with ACL injuries presented during the study period, of whom 61 met inclusion criteria. These were compared with 61 age-matched ACL-intact controls (mean age 32.0±8.6 years vs 30.3±8.6 years). Forty (65.57%) ACL-injured patients and 39 (63.93%) controls were male (Table 1).

**Table 1:** Demographic distribution of ACL injured and ACL intact groups.

	ACL injured (n=61)	ACL Intact (n=61)
Mean Age	30.3±8.63	32.0±8.6
Gendern n(%)		
Male	40(65.57)	39(63.93)
Female	21(34.43)	22(36.07)

ACL: Anterior Cruciate Ligament.

**Table 2:** Comparison of anatomical parameters between ACL injured and ACL intact groups (mean±SD).

Parameters	ACL injured (n=61)	ACL intact (n=61)	p-value
NW coronal (cm)	1.90±0.28	2.00±0.27	.04^*^
NW axial (cm)	1.98±0.30	2.14±0.33	<.01^*^
NWI coronal	0.27±0.03	0.29±0.03	.01^*^
NWI axial	0.28±0.04	0.30±0.04	<.01^*^
NA coronal (degrees)¥	49.89±6.12	53.47±6.52	<.01^*^
NA axial (degrees)	48.70±6.67	51.83±6.74	.01^*^
PTS lateral (degrees)	11.24±5.33	8.76±4.94	.01^*^
PTS medial (degrees)	11.23±5.21	8.76±4.76	.01^*^

* p<0.05 statistically significant.

¥ Primary parameter used for sample size calculation.

ACL: Anterior Cruciate Ligament, NA: Notch Angle, NW: Notch Width, NWI: Notch Width Index, PTS: Posterior Tibial Slope.

**Table 3:** Distribution of notch shapes in ACL injured and ACL intact groups.

Notch shape	ACL injured n(%)	ACL intact n(%)
Type A	33(54.10%)	26(42.62%)
Type U	26(42.62%)	32(52.46%)
Type W	2(3.28%)	3(4.92%)

ACL: Anterior Cruciate Ligament.

In our population, the ACL-injured group had smaller femoral notch dimensions compared to controls, with coronal width measuring 1.90±0.28 cm versus 2.00±0.27 cm and axial width 1.98±0.30 cm versus 2.14±0.33 cm. Notch width indices were consistently lower in injured patients, measuring 0.27±0.03 versus 0.29±0.03 in the coronal plane and 0.28±0.04 versus 0.30±0.04 axially. Additionally, ACL-injured patients had more acute notch angles in both coronal (49.89±6.12° vs 53.47±6.52°) and axial (48.70±6.67° vs 51.83±6.74°) planes. Steeper posterior tibial slopes were observed in ACL-injured patients, with lateral slopes measuring 11.24±5.33° versus 8.76±4.94° and medial slopes 11.23±5.21° versus 8.76±4.76° (Table 2).

Notch shape distribution differed between groups, with Type A morphology present in 33 (54.10%), Type U was observed in 26 (42.62%) injured patients compared to 26 (42.62%) and 32 (52.46%) intact knees respectively (Table 3).

## DISCUSSION

Two thirds of our ACL injured cohort was male (65.6%), though this cross-sectional study design does not allow determination of the incidence. The finding contrasts with literature reports suggesting increased incidence of ACL injury in females (2.49.5-fold) mostly attributed to anatomic factors (narrow notches, wider pelvis) and neuromuscular factor (H:Q ratio, laxity).^5^ The male predominance could be attributed to non-random convenience case selection and also to socioeconomic biases in low resources setting on reporting and seeking care.

We found that the ACL-injured group had smaller femoral notch dimensions and lower notch width indices compared to controls in both coronal and axial measurements. Our findings are consistent with the findings by Basukala et al.^8^ (Nepalese population) and Hoteya et al.^7^ Narrow notches house thinner and weaker ACLs has also been shown by our co-author in another study (Nepalese Population).^11,12^ Impingement is greater in narrow notches; but controversy exists whether it is the geometry or the volume of ACL or amalgamation of these characteristics that predispose to ACL injury.^3^ According to Hirtler, Rohrich and Kainberger, the intercondylar notch changes throughout life, narrowing more distally and widening more proximally.^2^ Studies have shown that African Americans tend to have wider and taller intercondylar notches and larger intercondylar notch angles than the Caucasians.^13^ It may be assumed that smaller persons have smaller femoral intercondylar notch, simply as a function of body size.

Variations in notch width occur depending on the sex, race and age.^6,13^ Therefore, a standard ratio called notch width index was taken as a standard to identify the risk factors for ACL injury.^13^ Hoteya et al proposed a cutoff value for coronal NWI of 0.25 as risk factor for ACL injury.^7^ The femoral notch width and notch width index findings in our study are in line with the findings of previous studies. Alentorn-Geli E et al had a contradictory finding in NWI compared to our study.^14^

The notch angles are interlinked with notch shapes.^14^ The injured groups in our study showed more acute notch angles (Table 2), suggesting a likely anatomic predisposition to ACL injury. Narrow notch angle leads to impingement of the ACL during anterior tibial translation and valgus stress on the knee.^14^ Our findings were in line with previous studies where a significant difference was noted in the notch angle measurements.^9,14^

Our study demonstrated steeper posterior tibial slopes, both medial and lateral, in ACL-injured patients, with lateral PTS (11.24±5.33° vs 8.76±4.94°) and medial PTS (11.23±5.21° vs 8.76±4.76°) (Table 2). Our study suggests that both medial and lateral PTS contribute to the risk for ACL injury. Prior study consistently suggests increased lateral slope as a plausible risk factor.^4,14,15^ For every 10 degrees increase in PTS, 6 mm of greater anterior tibial translation was noted in ACL injured knees by Dejour and Bonnin.^15^ Maroune et al found elevated PTS to increase ACL loading during mid stance gait.^16^ Our finding are on medial PTS are consistent with study by Alentorn-Geli E et al, where they found a very strong correlation with ACL injury (p<.001).^14^ The role of medial PTS is still a subject of debate with minimal to no association across various studies.^4,17,18^ However, Alentorn-Geli E et al. et al emphasized this association to be more evident among male patients.^14^ Male predominance in our study could be a plausible cause. It also highlights the need of further investigation especially among male patients.

Although the differences in various parameter shown in table 2 and discussed previously are statistically significant, the absolute differences are often a few millimeters or few degrees. The interpretation of such subtle differences, though measurable, may need stringent caution as their clinical significance may be limited and corrections of this magnitude are rarely achievable with surgical precision.

Type A notch morphology was more common in ACL-injured knees than non-injured ones (Table 3). While a Nepalese study found Type A notches in 74% of ACL injured and correlated with ACL injury (p<0.001), our study showed no such association (p = 0.94).^8^ Both studies used convenience sampling method with limited sample size and further smaller subgroups, selection bias could be an important reason for these differences. Failure to differentiate the underlying populations (athletes or non-athletes) and mode of injury (trauma and sports, contact and non-contact) in both studies may be attributed to this discrepancy. The debate on the notch type (A/U/W) persists on which type predisposes to ACL injury, though narrow morphology consistently shows higher risk.^6,9^ Standardization of classification system and robust study designs addressing the confounding factors are needed to establish definitive risk association.

Though computed tomography (CT) Scan could have demonstrated the bony parameter with better accuracy, MRI are gold standard for ACL injury diagnosis, avoiding additional cost and radiation hazard, and it also provides sufficient detail of bony anatomy relevant to the study’s objectives. While the sample size taken was adequate to detect clinically significant differences in lateral PTS and coronal notch angles, it may have been insufficient to identify smaller anatomical variations, such as those in notch width index and medial PTS. Moreover, our study could not quantify a narrow notch and does not give a definitive cutoff value, giving no added benefit in clinical decision making. The absence of quantified interobserver reliability metrics questions the strength of measurement precision. Additionally, the multiple comparisons performed across anatomical parameters increase the risk of type I errors. The potential for meaningful discussion on sexual dimorphism is limited by the gender imbalance in the cohort, with a disproportionate number of male participants. Use of convenience sampling could have introduced selection bias limiting the inclusion of significant case. As a single-center, cross-sectional study, these findings may lack generalizability to broader populations.

Although we matched age and sex between groups, femoral anatomy is also influenced by height, ethnicity and several other confounders not controlled in this study. Future studies controlling these parameters could add greater relevance. Similarly, while our findings are statistically significant, the small effect size raises a valid question about their clinical significance. Further research with larger sample sizes and robust study design are essential to evaluate whether these subtle anatomical differences meaningfully translate into real-world clinical outcomes.

## CONCLUSIONS

Our study confirms that narrower femoral notch dimensions (smaller notch width, lower notch width index, and more acute notch angles) and increased posterior tibial slope (both medial and lateral) are significantly associated with ACL injury risk. These findings underscore the importance of anatomical factors as possible risk factors for ACL injury and highlight the need for individualized risk assessment, particularly in at-risk populations.

## Data Availability

The data are available from the corresponding author upon reasonable request.
